# Aqua­[4-(hy­droxy­imino­meth­yl)pyridine-κ*N* ^1^](imino­diacetato-κ^3^ *O*,*N*,*O*′)copper(II)

**DOI:** 10.1107/S1600536811033459

**Published:** 2011-08-27

**Authors:** Yisheng Yang, Wenxiang Chai, Li Song, Yunyun Yang, Jiongke Chen

**Affiliations:** a College of Materials Science and Engineering, China Jiliang University, Hangzhou 310018, People’s Republic of China; b Department of Chemistry, Key Laboratory of Advanced Textile Materials and, Manufacturing Technology of Education Ministry, Zhejiang Sci-Tech University, Hangzhou 310018, People’s Republic of China

## Abstract

In the title complex, [Cu(C_4_H_5_NO_4_)(C_6_H_6_N_2_O)(H_2_O)], conventionally abbreviated Cu(IDA)(4-OXPy)(H_2_O), where IDA is imino­diacetate and 4-OXPy is 4-(hy­droxy­imino­meth­yl)pyridine, the Cu^II^ atom exhibits a distorted square-pyramidal coordination geometry, which is constructed from two O atoms and one N atom from a IDA ligand, one N atom from 4-OXPy ligand and one O atom from water. This mol­ecule looks like a space shuttle, the IDA ligand is its empennage (tail), and the 4-OXPy ligand is its airframe. The complexes are linked into two-dimensional supra­molecular layers parallel to (100) by three pairs of O—H⋯O hydrogen bonds. Two pairs of N—H⋯O hydrogen bonds further connect these supra­molecular layers, forming a three-dimensional supra­molecular network.

## Related literature

For related ternary complexes of copper(II), IDA and an *N*-heterocyclic ligand, see: Roman-Alpiste *et al.* (1999 ▶); Kundu *et al.* (2005 ▶); Chen *et al.* (1990 ▶); Zhang *et al.* (2008 ▶); Selvakumar *et al.* (2006 ▶); Siddiqi *et al.* (2009 ▶); Setha *et al.* (2010 ▶); Campos *et al.* (1996 ▶); Castineiras *et al.* (1995 ▶); Brandi-Blanco *et al.* (2003 ▶); Craven *et al.* (2003 ▶). For hydrogen bonding, see: Desiraju & Steiner (1999 ▶). For the *PLATON* program, see: Spek (2009 ▶).
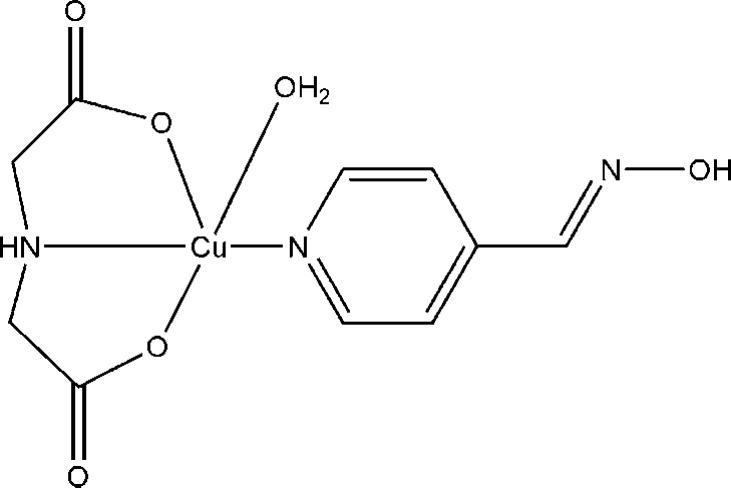


## Experimental

### 

#### Crystal data

[Cu(C_4_H_5_NO_4_)(C_6_H_6_N_2_O)(H_2_O)]
*M* *_r_* = 334.78
Triclinic, 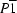

*a* = 5.520 (7) Å
*b* = 6.715 (9) Å
*c* = 17.21 (2) Å
α = 93.41 (2)°
β = 93.952 (13)°
γ = 106.52 (2)°
*V* = 608.1 (13) Å^3^
*Z* = 2
Mo *K*α radiation
μ = 1.83 mm^−1^
*T* = 293 K
0.10 × 0.10 × 0.10 mm

#### Data collection

Rigaku R-AXIS RAPID diffractometer
Absorption correction: multi-scan (*ABSCOR*; Higashi, 1995 ▶) *T*_min_ = 0.838, *T*_max_ = 0.838
4742 measured reflections
2739 independent reflections
2269 reflections with *I* > 2σ(*I*)
*R*_int_ = 0.036

#### Refinement

*R*[*F* ^2^ > 2σ(*F* ^2^)] = 0.041
*wR*(*F* ^2^) = 0.095
*S* = 1.04
2739 reflections
229 parameters
3 restraints
H atoms treated by a mixture of independent and constrained refinement
Δρ_max_ = 0.57 e Å^−3^
Δρ_min_ = −0.50 e Å^−3^

### 

Data collection: *PROCESS-AUTO* (Rigaku, 1998 ▶); cell refinement: *PROCESS-AUTO*; data reduction: *CrystalStructure* (Rigaku/MSC, 2004 ▶); program(s) used to solve structure: *SHELXS97* (Sheldrick, 2008 ▶); program(s) used to refine structure: *SHELXL97* (Sheldrick, 2008 ▶); molecular graphics: *SHELXTL* (Sheldrick, 2008 ▶); software used to prepare material for publication: *SHELXTL*.

## Supplementary Material

Crystal structure: contains datablock(s) I, global. DOI: 10.1107/S1600536811033459/zk2022sup1.cif

Structure factors: contains datablock(s) I. DOI: 10.1107/S1600536811033459/zk2022Isup2.hkl

Additional supplementary materials:  crystallographic information; 3D view; checkCIF report

## Figures and Tables

**Table 1:** Hydrogen-bond geometry (Å, °)

*D*—H⋯*A*	*D*—H	H⋯*A*	*D*⋯*A*	*D*—H⋯*A*
O5—H5*A*⋯O2^i^	0.82 (2)	1.99 (2)	2.771 (4)	159 (3)
O6—H6*A*⋯O3^ii^	0.82 (2)	1.97 (3)	2.694 (4)	147 (3)
O5—H5*B*⋯O3^iii^	0.82 (2)	1.99 (2)	2.796 (4)	172 (4)
N1—H1⋯O1^iv^	0.83 (3)	2.59 (4)	3.214 (5)	133 (3)
N1—H1⋯O2^iv^	0.83 (3)	2.42 (3)	3.026 (4)	130 (3)

Symmetry codes: (i) 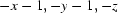; (ii) 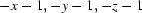; (iii) 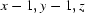; (iv) 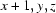; (v) 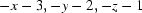.

**Fig. 1.**
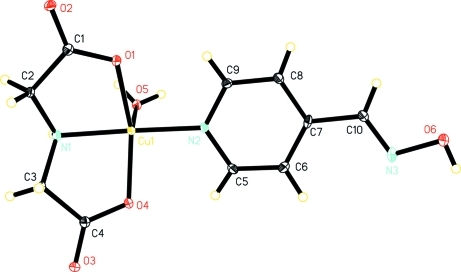
A view of the structure of I, showing the atom-numbering scheme. Displacement ellipsoids are drawn at the 30% probabilithy level.

**Fig. 2.**
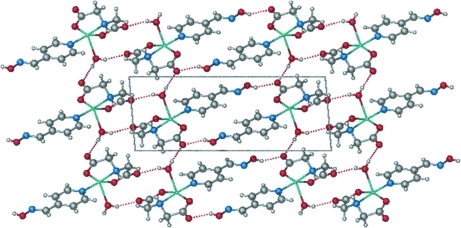
The supramolecular organic-inorganic hybrid layer constructed by hydrogen bonds, viewed along the a-direction.

**Fig. 3.**
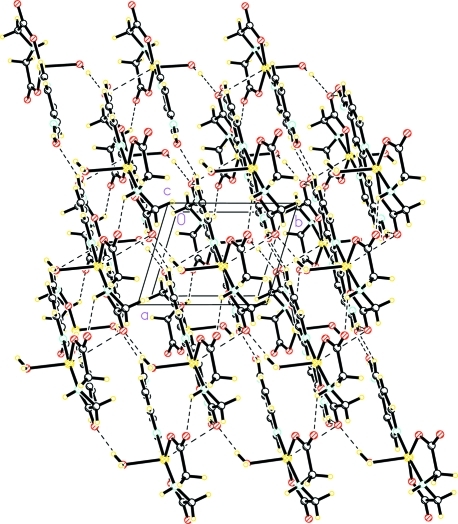
The packing diagram of I, viewed along the c-direction.

## supplementary crystallographic information

### Comment

Due to the tridentate chelating function of the iminodiacetate anion and the plasticity of the copper(ii) coordination stereochemistry, an important research has been devoted to the structure of [Cu(IDA)(H_2_)_2_]_n_ (Roman-Alpiste *et al.*, 1999) and a variety of mixed-ligand complexes of copper(ii), IDA and N-heterocyclic donor (auxiliary). These ternary complexes are a source of inorganic structural corelations and bioinorganic model compounds for mono- or di-nuclear copper proteins. Here, we report one of those ternary complexes Cu(IDA)(4-OXPy)(H_2_O) (I), composed from copper(ii), IDA and 4-OXPy ligand.

In compound (I), the copper(ii) exhibits distorted square pyramid coordination geometry, which is constructed from two O atoms and one N atom from a IDA ligand, one N atom from 4-OXPy ligand and other one O atom from water. In this CuO_3_N_2_ square pyramid, the Cu1—O1 = 1.998 (3) Å, Cu1—O4 = 1.969 (3) Å, Cu1—O5 = 2.326 (4) Å, Cu1—N1 = 1.998 (3) Å, Cu1—N2 = 1.968 (3) Å. All bond lengths are within commonly accepted values in the literature (Roman-Alpiste *et al.*, 1999; Kundu *et al.*, 2005; Chen *et al.*, 1990; Zhang *et al.*, 2008). This molecule looks like a space shuttle, the IDA ligand just as its empennage, and the 4-OXPy ligand as its airframe. And by virtue of three pairs of O—H···O and two pairs of N—H···O hydrogen bonds, the complexes are linked into a three-dimensional supramolecular network. The hydrogen bonding data are in the range of standard examples (Desiraju *et al.*, 1999) and have been examined by the *PLATON* program (Spek, 2009). Firstly, along the crystal plane group of {1 - 1 0}, the adjacent molecules connect each other through three pairs of O—H···O hydrogen bonds [O5—H5A···O2, O5—H5B···O3 and O6—H6A···O3] (details listed in Table 2) to form supramolecular layers. And then, the neighbouring layers fuse one by one *via* other two pairs of N—H···O hydrogen bonds [N1—H1···O1 and N1—H1···O2] (details listed in Table 2) to give out a 3-D supramolecular network.

### Experimental

The title compound (I) was synthesized by solution reaction of Cu_2_(OH)_2_CO_3_ (23 mg, 0.1 mmol), H_2_IDA (27 mg, 0.2 mmol), and 4-AOXPy (25 mg, 0.2 mmol) in 15 ml water at room temperature. The subsequent solution was filtered and placed for evaperation. After several days, the blue crystals of (I) were obtained in a yield of 91% (61 mg). Anal.Calc. for C_10_H_13_CuN_3_O_6_ (%): C, 35.88; H, 3.91; N, 12.55; O, 28.67. Found: C, 35.53; H, 3.66; N, 12.87; O, 28.98. Crystals of (I) suitable for single-crystal X-ray diffraction were selected directly from the sample as prepared.

### Refinement

A suitable single-crystal of (I) was selected and mounted on a thin glass fiber with the aid of an epoxy resin. The XRD data were collected with Ω scan mode at 293 (2) K on a Rigaku RAXIS-RAPID CCD diffractometer (Mo Kα, λ = 0.71075 Å). The structure was solved using direct methods and refined by full-matrix least-squares techniques. All non-hydrogen atoms were assigned anisotropic displacement parameters in the refinement. All hydrogen atoms were picked out from difference Fourier peaks and restrained the distances as 0.82 (2) Å on the O—H bonds. The structure was refined on F2 using *SHELXTL97* software package (Sheldrick *et al.*, 2008) without any unusual events.

### Crystal data

**Table:**

[Cu(C_4_H_5_NO_4_)(C_6_H_6_N_2_O)(H_2_O)]	*V* = 608.1 (13) Å^3^
*M**_r_* = 334.78	*Z* = 2
Triclinic, *P*1	*F*(000) = 342
Hall symbol: -P 1	*D*_x_ = 1.829 Mg m^−^^3^
*a* = 5.520 (7) Å	Mo *K*α radiation, λ = 0.71075 Å
*b* = 6.715 (9) Å	µ = 1.83 mm^−^^1^
*c* = 17.21 (2) Å	*T* = 293 K
α = 93.41 (2)°	Prism, blue
β = 93.952 (13)°	0.10 × 0.10 × 0.10 mm
γ = 106.52 (2)°	

### Data collection

**Table:**

Rigaku R-AXIS RAPID diffractometer	2739 independent reflections
Radiation source: fine-focus sealed tube	2269 reflections with *I* > 2σ(*I*)
graphite	*R*_int_ = 0.036
Detector resolution: 14.6306 pixels mm^-1^	θ_max_ = 27.5°, θ_min_ = 3.2°
CCD_Profile_fitting scans	*h* = −6→7
Absorption correction: multi-scan (*ABSCOR*; Higashi, 1995)	*k* = −8→6
*T*_min_ = 0.838, *T*_max_ = 0.838	*l* = −22→22
4742 measured reflections	

### Refinement

**Table:**

Refinement on *F*^2^	Primary atom site location: structure-invariant direct methods
Least-squares matrix: full	Secondary atom site location: difference Fourier map
*R*[*F*^2^ > 2σ(*F*^2^)] = 0.041	Hydrogen site location: inferred from neighbouring sites
*wR*(*F*^2^) = 0.095	H atoms treated by a mixture of independent and constrained refinement
*S* = 1.04	*w* = 1/[σ^2^(*F*_o_^2^) + (0.0435*P*)^2^] where *P* = (*F*_o_^2^ + 2*F*_c_^2^)/3
2739 reflections	(Δ/σ)_max_ < 0.001
229 parameters	Δρ_max_ = 0.57 e Å^−^^3^
3 restraints	Δρ_min_ = −0.50 e Å^−^^3^

### Special details

**Table:**

Geometry. All e.s.d.'s (except the e.s.d. in the dihedral angle between two l.s. planes) are estimated using the full covariance matrix. The cell e.s.d.'s are taken into account individually in the estimation of e.s.d.'s in distances, angles and torsion angles; correlations between e.s.d.'s in cell parameters are only used when they are defined by crystal symmetry. An approximate (isotropic) treatment of cell e.s.d.'s is used for estimating e.s.d.'s involving l.s. planes.
Refinement. Refinement of *F*^2^ against ALL reflections. The weighted *R*-factor *wR* and goodness of fit *S* are based on *F*^2^, conventional *R*-factors *R* are based on *F*, with *F* set to zero for negative *F*^2^. The threshold expression of *F*^2^ > σ(*F*^2^) is used only for calculating *R*-factors(gt) *etc*. and is not relevant to the choice of reflections for refinement. *R*-factors based on *F*^2^ are statistically about twice as large as those based on *F*, and *R*- factors based on ALL data will be even larger.

### Fractional atomic coordinates and isotropic or equivalent isotropic displacement parameters (Å^2^)

**Table:**

	*x*	*y*	*z*	*U*_iso_*/*U*_eq_	
Cu1	−0.37748 (7)	−0.42758 (6)	−0.194474 (19)	0.01297 (13)	
N1	−0.1348 (5)	−0.2773 (4)	−0.10405 (14)	0.0125 (5)	
O1	−0.6388 (4)	−0.4320 (3)	−0.11931 (11)	0.0145 (4)	
O2	−0.6897 (4)	−0.3155 (3)	0.00069 (12)	0.0169 (5)	
O3	0.2937 (4)	−0.0787 (3)	−0.24552 (12)	0.0178 (5)	
O4	−0.0910 (4)	−0.3095 (3)	−0.25623 (11)	0.0154 (5)	
O5	−0.3355 (4)	−0.7481 (3)	−0.16195 (12)	0.0165 (5)	
N2	−0.6174 (5)	−0.5564 (4)	−0.28578 (13)	0.0113 (5)	
C7	−0.9679 (6)	−0.7319 (5)	−0.41322 (16)	0.0146 (6)	
O6	−1.3312 (5)	−0.8857 (4)	−0.59936 (13)	0.0241 (5)	
C1	−0.5549 (6)	−0.3314 (4)	−0.05274 (16)	0.0133 (6)	
C4	0.0914 (6)	−0.1689 (4)	−0.21843 (16)	0.0131 (6)	
N3	−1.1134 (5)	−0.7984 (4)	−0.54871 (15)	0.0181 (6)	
C3	0.0505 (6)	−0.1025 (5)	−0.13563 (17)	0.0126 (6)	
C5	−0.5516 (6)	−0.5396 (5)	−0.35971 (17)	0.0154 (6)	
C9	−0.8561 (6)	−0.6636 (5)	−0.27556 (17)	0.0146 (6)	
C2	−0.2717 (6)	−0.2199 (5)	−0.03983 (16)	0.0127 (6)	
C6	−0.7194 (6)	−0.6254 (5)	−0.42368 (17)	0.0176 (7)	
C8	−1.0364 (6)	−0.7541 (5)	−0.33692 (17)	0.0149 (6)	
C10	−1.1658 (6)	−0.8162 (5)	−0.47803 (18)	0.0191 (7)	
H2B	−0.253 (7)	−0.070 (6)	−0.038 (2)	0.026 (5)*	
H9	−0.894 (6)	−0.675 (5)	−0.2254 (19)	0.013 (8)*	
H6	−0.668 (7)	−0.608 (6)	−0.475 (2)	0.029 (10)*	
H3B	0.201 (7)	−0.051 (6)	−0.102 (2)	0.026 (5)*	
H5	−0.393 (7)	−0.473 (5)	−0.3613 (18)	0.012 (8)*	
H3A	−0.027 (6)	0.015 (5)	−0.1426 (19)	0.020 (9)*	
H8	−1.188 (7)	−0.826 (5)	−0.3240 (19)	0.023 (10)*	
H10	−1.331 (7)	−0.874 (5)	−0.4651 (19)	0.023 (9)*	
H2A	−0.204 (6)	−0.249 (5)	0.0091 (19)	0.015 (8)*	
H1	−0.062 (6)	−0.363 (5)	−0.0895 (19)	0.014 (9)*	
H5A	−0.347 (7)	−0.761 (6)	−0.1154 (11)	0.026 (5)*	
H5B	−0.439 (6)	−0.853 (4)	−0.1834 (19)	0.026 (5)*	
H6A	−1.267 (6)	−0.858 (6)	−0.6405 (14)	0.026 (5)*	

### Atomic displacement parameters (Å^2^)

**Table:**

	*U*^11^	*U*^22^	*U*^33^	*U*^12^	*U*^13^	*U*^23^
Cu1	0.0110 (2)	0.0155 (2)	0.00976 (18)	0.00041 (15)	−0.00023 (13)	−0.00174 (13)
N1	0.0124 (13)	0.0124 (12)	0.0122 (12)	0.0029 (11)	0.0000 (10)	0.0005 (10)
O1	0.0120 (11)	0.0191 (11)	0.0104 (9)	0.0021 (9)	−0.0006 (8)	−0.0005 (8)
O2	0.0117 (11)	0.0267 (12)	0.0123 (10)	0.0058 (9)	0.0020 (8)	0.0007 (9)
O3	0.0161 (12)	0.0192 (11)	0.0139 (10)	−0.0022 (9)	0.0037 (9)	0.0010 (9)
O4	0.0123 (11)	0.0178 (11)	0.0123 (10)	−0.0011 (9)	0.0006 (8)	−0.0020 (8)
O5	0.0169 (12)	0.0153 (11)	0.0138 (10)	−0.0002 (9)	0.0007 (9)	−0.0006 (9)
N2	0.0088 (12)	0.0129 (12)	0.0115 (11)	0.0025 (10)	−0.0004 (9)	−0.0007 (9)
C7	0.0210 (17)	0.0124 (14)	0.0092 (13)	0.0049 (12)	−0.0040 (12)	−0.0020 (11)
O6	0.0184 (13)	0.0336 (14)	0.0139 (11)	−0.0012 (11)	−0.0008 (9)	−0.0020 (10)
C1	0.0140 (16)	0.0145 (15)	0.0123 (13)	0.0055 (12)	−0.0009 (11)	0.0039 (11)
C4	0.0138 (15)	0.0130 (14)	0.0132 (13)	0.0052 (12)	−0.0003 (11)	0.0018 (11)
N3	0.0179 (14)	0.0198 (14)	0.0161 (12)	0.0074 (11)	−0.0063 (11)	−0.0022 (11)
C3	0.0127 (15)	0.0127 (14)	0.0097 (13)	0.0002 (12)	−0.0002 (11)	−0.0024 (11)
C5	0.0105 (16)	0.0176 (15)	0.0158 (14)	0.0002 (13)	−0.0001 (12)	0.0028 (12)
C9	0.0154 (16)	0.0147 (15)	0.0122 (14)	0.0015 (12)	0.0043 (12)	−0.0005 (11)
C2	0.0123 (15)	0.0134 (15)	0.0108 (13)	0.0021 (12)	0.0012 (11)	−0.0022 (11)
C6	0.0192 (17)	0.0204 (16)	0.0115 (14)	0.0030 (13)	0.0027 (12)	0.0005 (12)
C8	0.0113 (16)	0.0159 (15)	0.0157 (14)	0.0017 (13)	0.0007 (12)	−0.0009 (12)
C10	0.0123 (17)	0.0252 (17)	0.0157 (15)	−0.0002 (14)	0.0005 (12)	−0.0014 (13)

### Geometric parameters (Å, °)

**Table:**

Cu1—N2	1.968 (3)	C7—C10	1.471 (4)
Cu1—O4	1.969 (3)	O6—N3	1.394 (4)
Cu1—O1	1.998 (3)	O6—H6A	0.822 (18)
Cu1—N1	1.998 (3)	C1—C2	1.523 (4)
Cu1—O5	2.326 (4)	C4—C3	1.519 (4)
N1—C2	1.474 (4)	N3—C10	1.274 (4)
N1—C3	1.479 (4)	C3—H3B	0.94 (4)
N1—H1	0.83 (3)	C3—H3A	1.01 (4)
O1—C1	1.281 (4)	C5—C6	1.372 (4)
O2—C1	1.241 (4)	C5—H5	0.87 (3)
O3—C4	1.246 (4)	C9—C8	1.382 (4)
O4—C4	1.274 (4)	C9—H9	0.90 (3)
O5—H5A	0.816 (18)	C2—H2B	0.98 (4)
O5—H5B	0.816 (18)	C2—H2A	0.95 (3)
N2—C9	1.339 (4)	C6—H6	0.95 (4)
N2—C5	1.349 (4)	C8—H8	0.89 (4)
C7—C6	1.385 (5)	C10—H10	0.93 (4)
C7—C8	1.396 (4)		
			
N2—Cu1—O4	94.86 (12)	O3—C4—O4	124.7 (3)
N2—Cu1—O1	96.38 (13)	O3—C4—C3	118.6 (3)
O4—Cu1—O1	158.10 (9)	O4—C4—C3	116.6 (3)
N2—Cu1—N1	175.97 (10)	C10—N3—O6	110.1 (3)
O4—Cu1—N1	83.76 (13)	N1—C3—C4	109.0 (2)
O1—Cu1—N1	83.72 (13)	N1—C3—H3B	112 (2)
N2—Cu1—O5	92.19 (11)	C4—C3—H3B	114 (2)
O4—Cu1—O5	105.52 (10)	N1—C3—H3A	109 (2)
O1—Cu1—O5	92.77 (9)	C4—C3—H3A	103.5 (19)
N1—Cu1—O5	91.83 (11)	H3B—C3—H3A	108 (3)
C2—N1—C3	115.9 (2)	N2—C5—C6	122.7 (3)
C2—N1—Cu1	110.8 (2)	N2—C5—H5	112 (2)
C3—N1—Cu1	106.36 (19)	C6—C5—H5	125 (2)
C2—N1—H1	109 (2)	N2—C9—C8	123.0 (3)
C3—N1—H1	109 (2)	N2—C9—H9	116 (2)
Cu1—N1—H1	105 (2)	C8—C9—H9	121 (2)
C1—O1—Cu1	115.4 (2)	N1—C2—C1	111.4 (2)
C4—O4—Cu1	114.2 (2)	N1—C2—H2B	110 (2)
Cu1—O5—H5A	111 (3)	C1—C2—H2B	107 (2)
Cu1—O5—H5B	117 (3)	N1—C2—H2A	111 (2)
H5A—O5—H5B	104 (4)	C1—C2—H2A	110 (2)
C9—N2—C5	117.8 (3)	H2B—C2—H2A	108 (3)
C9—N2—Cu1	119.8 (2)	C5—C6—C7	119.6 (3)
C5—N2—Cu1	122.4 (2)	C5—C6—H6	121 (2)
C6—C7—C8	118.2 (3)	C7—C6—H6	120 (2)
C6—C7—C10	123.6 (3)	C9—C8—C7	118.7 (3)
C8—C7—C10	118.2 (3)	C9—C8—H8	116 (2)
N3—O6—H6A	97 (3)	C7—C8—H8	125 (2)
O2—C1—O1	124.3 (3)	N3—C10—C7	120.6 (3)
O2—C1—C2	118.4 (3)	N3—C10—H10	122 (2)
O1—C1—C2	117.3 (3)	C7—C10—H10	117 (2)

### Hydrogen-bond geometry (Å, °)

**Table:**

*D*—H···*A*	*D*—H	H···*A*	*D*···*A*	*D*—H···*A*
C5—H5···O4	0.87 (3)	2.36 (3)	2.973 (4)	128 (3)
C9—H9···O1	0.90 (3)	2.44 (3)	3.014 (4)	121 (3)
O5—H5A···O2^i^	0.82 (2)	1.99 (2)	2.771 (4)	159 (3)
O6—H6A···O3^ii^	0.82 (2)	1.97 (3)	2.694 (4)	147 (3)
O5—H5B···O3^iii^	0.82 (2)	1.99 (2)	2.796 (4)	172 (4)
N1—H1···O1^iv^	0.83 (3)	2.59 (4)	3.214 (5)	133 (3)
N1—H1···O2^iv^	0.83 (3)	2.42 (3)	3.026 (4)	130 (3)
C10—H10···O6^v^	0.93 (4)	2.47 (4)	3.340 (5)	156 (3)

Symmetry codes: (i) −*x*−1, −*y*−1, −*z*; (ii) −*x*−1, −*y*−1, −*z*−1; (iii) *x*−1, *y*−1, *z*; (iv) *x*+1, *y*, *z*; (v) −*x*−3, −*y*−2, −*z*−1.
